# *NALCN* Promoter Methylation as a Biomarker for Metastatic Risk in a Cohort of Non-Small Cell Lung Cancer Patients

**DOI:** 10.3390/biom14121514

**Published:** 2024-11-27

**Authors:** Eleni Thanou, Dora Lontra, Ioanna Balgouranidou, Eleni Efthimiadou, Alexandra Delipetrou, Emilia Tsaroucha, Maria Theodosiou, Vassilis Georgoulias, Athanasios Kotsakis, Evi Lianidou, Athina Markou

**Affiliations:** 1 Analysis of Circulating Tumor Cells, Laboratory of Analytical Chemistry, Department of Chemistry, University of Athens, 15771 Athens, Greece; elenathanou99@gmail.com (E.T.); doralontra@hotmail.gr (D.L.); alexdelipetrou@gmail.com (A.D.); lianidou@chem.uoa.gr (E.L.); 2 Department of Medical Oncology, University General Hospital of Alexandroupolis, Medical School, Democritus University of Thrace, 68100 Alexandroupolis, Greece; ioannabalg@gmail.com; 3 Laboratory of Inorganic Chemistry, Department of Chemistry, National and Kapodistrian University of Athens, Panepistimiopolis-Zografou, 15771 Athens, Greece; efthim@chem.uoa.gr (E.E.); mtheodoss@chem.uoa.gr (M.T.); 4 8th Department of Pulmonary Diseases, “Sotiria” General Hospital, 11527 Athens, Greece; emilygeola@yahoo.gr; 5 First Department of Medical Oncology, Metropolitan General Hospital, 15562 Athens, Greece; georgulv@otenet.gr; 6 Department of Medical Oncology, University General Hospital of Larissa, 41334 Larissa, Greece; thankotsakis@hotmail.com

**Keywords:** NALCN, methylation, NSCLC, metastasis, prognostic biomarker

## Abstract

Liquid biopsy enables real-time monitoring of tumor development and response to therapy through the analysis of CTCs and ctDNA. NALCN is a sodium leak channel that is frequently involved in tumor evolution and immunity and acts as a tumor suppressor. Deletion of NALCN has been shown to increase cancer metastasis and the number of CTCs in peripheral blood. In this study, we investigated for the first time NALCN promoter methylation in (a) Aza-treated cell lines (A549, TE671, BT20, and MDA-MB-468), (b) paired NSCLC tissues (n = 22), and (c) plasma cell-free DNA (ctDNA) from patients with NSCLC (early stage n = 39, metastatic n = 39) and DNA from 10 healthy donors (HD) using a newly developed highly specific and sensitive real-time MSP method. Treatment with 5′-aza-dC induced the expression of NALCN only in the A549 cell line, suggesting that DNA methylation regulates its expression in certain cancers. The mRNA expression levels of NALCN were quantified in non-small cell lung cancer (NSCLC) and adjacent non-cancerous tissues, and it was found to be underexpressed in 54.5% of tumor tissues, with significantly higher expression in recurrence-free patients (*p* = 0.009) than in patients who relapsed. The NALCN methylation level was not statisticallysignificantlycorrelated with the corresponding expression (*p* = 0.439), while Kaplan–Meier analysis showed an association between NALCN promoter hypermethylation and worse disease-free intervals (DFIs) (*p* = 0.017). Evaluation of NALCN methylation in ctDNA revealed that it was detected in 5.1% of early and 10.2% of advanced cases. Our results strongly suggest that epigenetic inactivation of NALCN may be a predictor of metastasis in NSCLC. Our results should be validated in further studies based on a larger patient cohort to further investigate whether DNA methylation of the NALCN promoter could serve as a potential prognostic DNA methylation biomarker and predictor of metastasis in NSCLC.

## Simple Summary

Liquid biopsy allows regular monitoring of cancer progression and response to treatment by analyzing circulating tumor cells (CTCs) and circulating tumor DNA (ctDNA) in plasma. This is the first time that the methylation status of NALCN in NSCLC has beenexamined and correlated with its expression. Thus, the detection of NALCN promoter methylation in NSCLC tumor tissue provides significant prognostic information for patients with NSCLC. However, we strongly believe that the evaluation of NALCN promoter methylation in plasma CTDNA as a non-invasive circulating tumor biomarker should be further investigated in a large and well-defined patient cohort.

## 1. Introduction

Lung cancer (LC) is the most common cause of cancer death in men and women worldwide, with a relative 5-year survival rate of 25% [1], often due to late diagnosis and metastasis [2,3]. Non-small cell lung cancer (NSCLC) is the most important pathologic subtype of lung cancer and is responsible for the majority of all lung cancer cases [4]. Research in the field of NSCLC has discovered a considerable number of useful genetic driver mutations and translocations for the targeted therapy of NSCLC patients [5].

Liquid biopsy is considered a cutting-edge approach in personalized medicine as it offers the possibility to monitor tumor progression and patient follow-up in real time [6]. As a non-invasive method, liquid biopsy provides crucial information through the analysis of circulating tumor cells (CTCs), circulating tumor DNA (ctDNA), circulating miRNAs (cfmiRNAs), and extracellular vesicles (EVs) [7]. Remarkably, liquid biopsy has succeeded in elucidating the mechanisms of metastasis in lung cancer [8].

In particular, it has been shown that epigenetically oriented cancer research in lung cancer could lead to the discovery of new biomarkers in NSCLC since DNA methylation changes are frequently detected and play a central role in carcinogenesis, diagnosis, and prognosis due to the stability of biomarkers [9,10,11]. In 1999, Esteller et al. demonstrated that aberrant promoter hypermethylation of tumor suppressor genes can be found in the blood DNA of NSCLC patients [12]. It is now clear that methylation of tumor suppressor genes in serum or plasma samples and in the corresponding primary tumors could be used as prognostic and diagnostic biomarkers [13,14,15], and methylation rates are higher in tissues than in plasma samples. Our group has reported on the prognostic significance of DNA methylation markers in plasma ctDNA and tissue from NSCLC patients [8,16,17], and several other groups have detected DNA methylation of various genes in sputum and bronchoalveolar lavage samples [18,19,20].

NALCN, a sodium leak channel that has various CpG sites [21], was identified by Eric B. Rahrmann et al. as an important factor in the metastatic cascade, as its loss of function was associated with cancer metastasis. HOLE analysis, which usesa program for the analysis of the pore dimensions of the ion channel structural model, has shown that mutations that occur in advanced cancers and lead to loss of function of NALCN cause the greatest pore closure, suggesting that this channel may be a tumor suppressor. In addition, studies in mice have shown that deletion of NALCN increases cancer metastasis, while the channel controls CTCs [22]. Furthermore, in vivo experiments with prostate cancer have shown that the extent of PC-3-bone metastases depends on the bioavailability of NALCN in prostate cancer cells and that its expression in these cells increases with prostate cancer aggressiveness [23]. NALCN plays a central role in tumor immunity and prognosis and has potential therapeutic and diagnostic aspects [24]. Studies show that NALCN is present in various cancers, including glioblastoma, non-small cell lung cancer, pancreatic cancer, small cell lung cancer (SCLC), and tumor-derived endothelial cells [25,26,27]. Studies have alsorevealed the upregulation of NALCN in the malignant transformation of the human hepatocyte cell line [28]. Therefore, NALCN appears to be a promising tool for blocking metastasis. However, it is crucial to test this approach on human tissues and samples [22].

The aim of the present study was to investigate, for the first time, (a) the presence of methylation in the promoter of the *NALCN* gene, and (b) the prognostic significance of *NALCN* promoter methylation in NSCLC patients. To this end, we first analyzed the expression of *NALCN* mRNA in aza-treated cell lines and then examined the methylation status of the *NALCN* promoter in NSCLC tissues, adjacent non-cancerous tissues, and ctDNA, as well as in healthy individuals.

## 2. Materials and Methods

### 2.1. Cell Culture and Aza Treatment

A549, TE671, MDA-MB-468, and BT-20 cells were cultured in DMEM (Dulbecco’s Modified Eagle Medium-DMEM), supplemented with 10% *v*/*v* heat-inactivated Fetal Bovine Serum (FBS), 2mM L-glutamine, and 1% antibiotic-antimycotic solution (Gibco, Grand Island, NE, USA) and antibiotics (100 units/mL penicillin and 100 μg/mL streptomycin) at 37 °C in a 5% CO_2_ atmosphere.

All cell culture media were supplemented with 10% fetal bovine serum and 1% antibiotic-antimycotic solution (Gibco, Grand Island, NE, USA). All cell lines were incubated at 37 °C under 5% CO_2_. Azacytidine (5-Aza, Sigma-Aldrich, St Louis, MO, USA) was dissolved in sterile water. A549, TE671, MDA-MB-468, and BT-20 cells were incubated with 20μM 5-Aza for 24, 48 and 72 h. Untreated cells with 5-Aza-dC were considered control cells.

### 2.2. Clinical Samples

Four different groups of clinical samples were analyzed:

(a) Primary NSCLC (fresh-frozen) tissues and corresponding adjacent non-neoplastic tissues from 22 patients, diagnosed with operable (stage I–III) NSCLC. All patients were treatment naïve when the samples were collected but after surgery, all patients received standard chemotherapy protocols for adjuvant NSCLC. A clinical relapse was documented in 12/22 (55%) patients during the follow-up period.

(b) Plasma samples from 39 patients with operable NSCLC, obtained before surgery.

(c) Plasma samples from 39 patients with advanced NSCLC, collected before the initiation of first-line chemotherapy.

(d) Plasma samples from 10 healthy blood donors (HD). All HD had no known illness or fever at the time of blood drawing and were ≥35 years old.

The clinicopathological characteristics of all patients are shown in Table 1.

The study was conducted in accordance with the 1964 Declaration of Helsinki and was approved by the ethics and scientific committee of the ‘Sotiria’ General Hospital for Chest Diseases, Athens, and also by the ethics and scientific committee of the University General Hospital of Alexandroupolis. All participating patients signed an informed consent form to participate in the study.

### 2.3. Isolation of Genomic DNA from Fresh-Frozen Primary Tumor Tissues

The manufacturer’s instructions were followed to isolate gDNA from fresh-frozen samples of NSCLC and corresponding adjacent tissues using the Qiagen DNeasy Blood and Tissue Kit (Hilden, Germany). Tissue sections containing >80% of tumor cells were used for DNA extraction and real-time methylation-specific PCR (real-time MSP) analysis.

### 2.4. Isolation of Plasma-cfDNA

After sample collection, plasma (in EDTA) was isolated from peripheral blood by centrifugation at 530× *g* for 10 min at room temperature within 2 h to 4 h. Upon separation, plasma samples were centrifuged again at 2000× *g* for 10 min before being transferred into 2 mL tubes and frozen at −70 °C until processing. According to previous research, ctDNA was extracted from 2.00 mL plasma using the QIAamp^®^ Circulating Nucleic Acid Kit 50 (Qiagen^®^, Hilden, Germany) [8].

### 2.5. Sodium Bisulfite Conversion

As previously described [8,16], ctDNA and gDNA from tissues were chemically modified with sodium bisulfite (SB). Negative and positive controls were included in each SB-reaction. A fully methylated (100%) positive control was used, namely the Universal Methylated Human DNA Standard (ZYMO Research, Orange, CA, USA). To preserve the SB-converted DNA samples, they will be stored at 70 °C until use. ACTB, an indicator of methylation, was assessed by real-time methylation-specific PCR after SB treatment; only samples amplified by MSP were studied.

### 2.6. RNA Extraction and cDNA Synthesis

The extraction of total RNA was carried out from 1 mL of cell lines diluted in Trizol, using the Qiagen RNeasy Mini Reagent Kit (Qiagen, Hilden, Germany) as per the manufacturer’s instructions. The elution volume was set at 10 μL in RNase-free water. All RNA preparation and handling processes were performed in a laminar flow hood under RNase-free conditions, with the isolated RNA stored at −80 °C until it was needed. cDNA synthesis was conducted using 500 ng of the isolated total RNA and the engineered M-MLV Reverse Transcriptase Basic Kit, 50 reactions (EnzyQuest P.C., Heraklion Crete), following the manufacturer’s guidelines.

### 2.7. InSilico Primers and Probes Design

For the design of all primers and probes from scratch, we utilized Wanderer (http://maplab.imppc.org/wanderer/, accessed on 1 December 2023), an interactive web platform for TCGA data, to identify all significantly methylated cytosine guanines (CGs) within the NALCN gene regions, based on the Illumina 450K analysis data. This approach aimed to incorporate as many CGs as feasible into the insilico design. Following the selection of the relevant gene region, we employed Primer Premier 5 software (Premier Biosoft International, San Francisco, CA, USA) for the insilico design of primers and probes, ensuring the avoidance of stable hairpin structures, primer dimers, cross-dimers, and false priming sites. The specificity of the sequences designed in silico was confirmed using the BLAST tool. (https://blast.ncbi.nlm.nih.gov/Blast.cgi, accessed on 1 December 2023). The sequences of primers and probes are available in Supplementary Table S1.

### 2.8. RT-qPCR Assay for NALCN mRNA Expression

For the evaluation of NALCN mRNA expression, a sensitive and specific real-time assay was conducted. Optimization experiments were performed, which included annealing temperature, time, primer pair concentrations, and the concentrations of buffer, MgCl_2_, dNTPs, and BSA The optimized protocol is thoroughly detailed in Supplementary Table S2. The reference gene utilized was Beta-2 microglobulin (B2M) as B2M is very highly expressed and small variations in expression between samples do not have a detectable effect on overall expression. The RT-qPCR was performed on the Mic qPCR Cycler (Bio Molecular Systems, Unit 5/3 Northward Street, Upper Coomera, QLD 4209, Australia).

### 2.9. Development and Analytical Validation of the Real-Time MSP Assay for NALCN Promoter Methylation

The experimental conditions for real-time methylation-specific PCR (MSP) targeting the NALCN promoter were initially optimized, and this refined assay was subsequently applied to both cell lines and clinical samples. A detailed description of the optimized protocol can be found in Supplementary Table S3.

To assess the analytical specificity of the NALCN real-time MSP, genomic DNA (gDNA) that had not undergone conversion, as well as SB-modified human placental gDNA samples that were unmethylated, were utilized; no amplification of the NALCN sequence was detected. Conversely, amplification was observed exclusively when SB-treated DNA from a fully methylated standard was employed (Supplementary Figure S1).

The analytical sensitivity of the established real-time MSP for *NALCN* was assessed using synthetic mixtures derived from serial dilutions of SB-converted DNA control samples, which included both 0% and 100% methylation levels along with varying percentages of methylation (0.5%, 5%, and 50%). The developed real-time MSP assay demonstrated the capability to specifically and reliably identify the presence of the 0.5% methylated NALCN sequence amidst a background of 99% unmethylated *NALCN* sequence (Supplementary Figure S2).

### 2.10. Statistical Analysis

Statistical analyses were performed using SPSS Statistics 26.0 (IBM Corp., Armonk, NY, USA). Expression values of *NALCN* were normalized to *B2M*. ΔCq values were calculated by using Cq values for *NALCN* and the corresponding B2M for each sample. We calculated ΔΔCq values using ΔCq values for cancerous samples and the mean value of ΔCq for normal samples (ΔΔCq = ΔCqcancer − Δcqnormal). Relative quantification (RQ) was based on the ΔΔCq method as described [25]. For paired tissue samples, ΔCq values were calculated as the differences between ΔCq values for each cancerous sample and its corresponding adjacent normal tissue. *NALCN* expression data are presented as fold change relative to the reference gene based on the formula of RQ = 2^−ΔΔCq^. Disease-free interval (DFI) and overall survival (OS) curves were calculated by using the Kaplan–Meier method and comparisons were performed using the log-rank test. *p*-values < 0.05 were considered statistically significant whereas a threshold was defined as a fold-change >2 for pair tissue samples. The Mann–Whitney U test was used in order to evaluate the differences in gene expression between different groups of samples. Spearman correlation analysis is used to explore the relationships among gene expression and hypermethylation of the same sample.

## 3. Results

**Figure 1:**
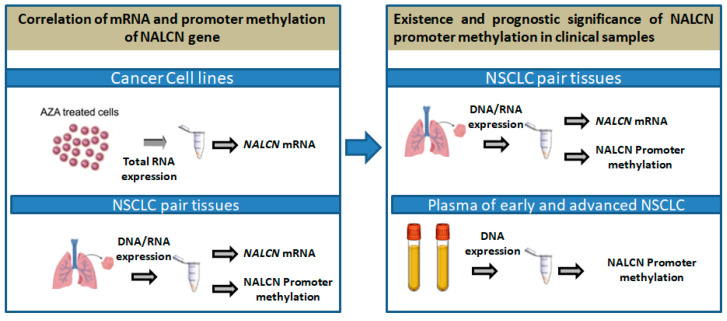
Outline of the experimental procedure.

The outline of the study is shown in Figure 1.

### 3.1. 5′-Aza-dC Treatment Induces NALCN Expression in A549 Cell Line

**Figure 2:**
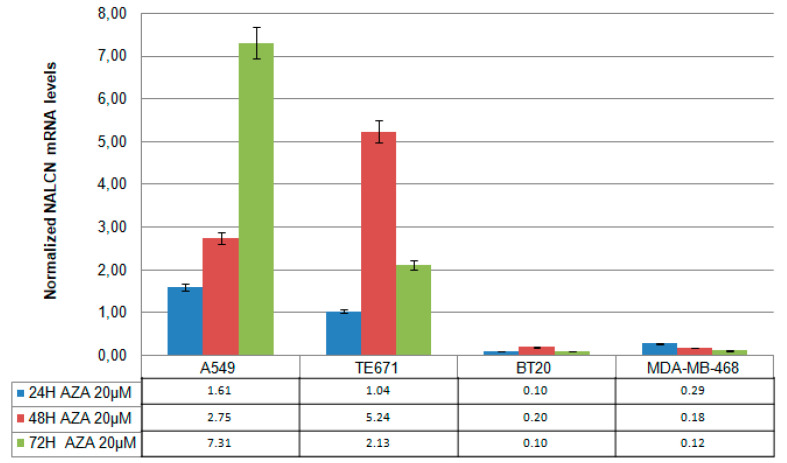
mRNA *NALCN* expression levels in aza-treated cell lines. Cell lines (A549, TE671, BT20, and MDA-MB-468) were treated with 20 µmol/L 5-Aza-dC for one, two, and three days. Each sample was subjected to qRT-PCR for detection of *NALCN* and *B2M* expression. Relative quantification was based on the ΔΔCq method.

To investigate whether the silencing function of NALCN is associated with promoter hypermethylation, the lung cell line (A549), cervical cell line (TE671), breast cell line (BT20), and MDA-MB-468 were treated with 5′-aza-dC (20 μm) for 24, 48, and 72 h, and NALCN expression levels were analyzed by RT-qPCR. Experiments were repeated three times independently and RT–qPCR results were normalized against B2M mRNA levels. In A549, NALCN mRNA expression was significantly induced after treatment with 20 μΜ 5′-aza-dC during days (Figure 2). Consistent with this, NALCN expression was also induced in ΤΕ671 cells after treatment with 20 μΜ 5′-aza-dC. In contrast, treatment with 5’-aza-dC did not alter the expression levels of NALCN in the two breast cancer cell lines tested, BT20 and MDA-MB-468 (Figure 2). Overall, these results suggest that DNA methylation regulatesthe NALCN expression in certain cancers, including NSCLC adenocarcinomas and meduloblastomas.

### 3.2. mRNA Expression and Promoter Methylation of NALCN in NSCLC Pair Tissues

**Figure 3:**
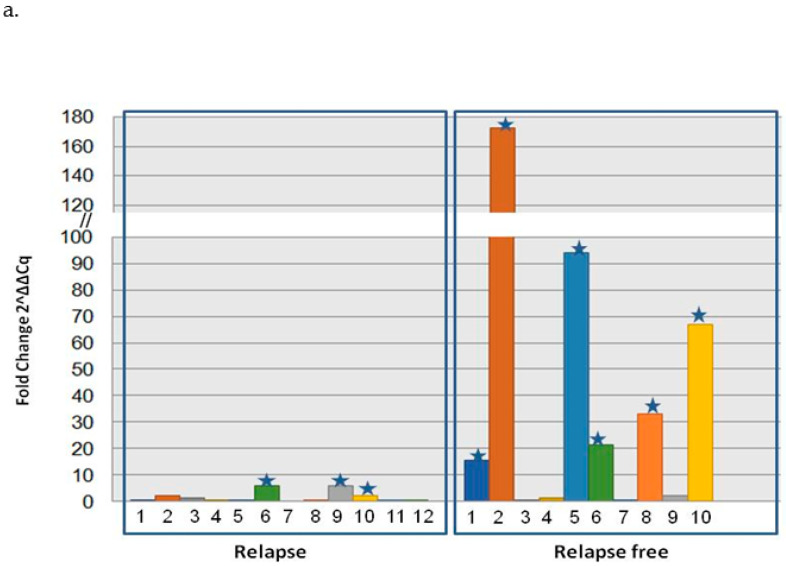

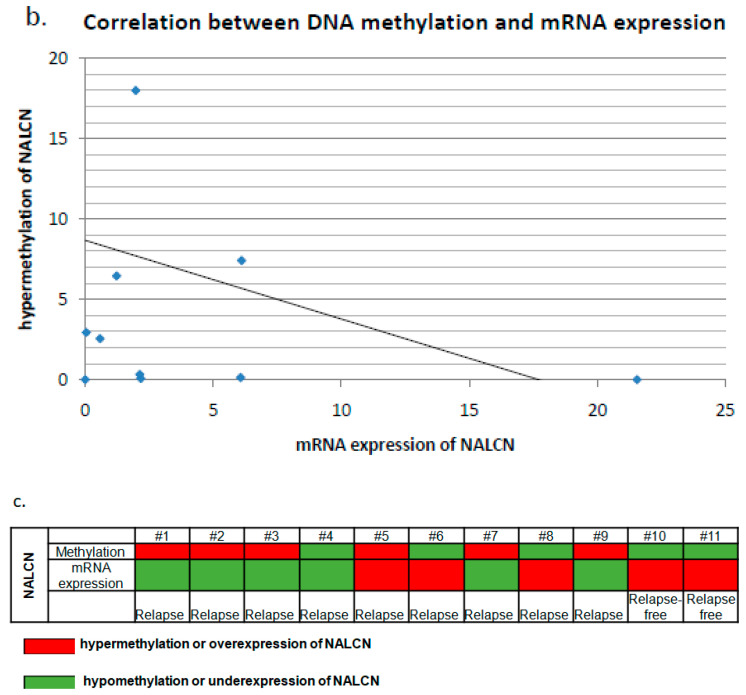
Correlation analysis of NALCN DNA methylation and mRNA gene expression in NLCLC pair tissues. (**a**) Relative expression levels of NALCN mRNA in paired adjacent normal tissues and cancerous tissues from NSCLC patients that were relapsed or were relapsed-free using quantitative PCR. Each number representsa pair of tissues of the same patient, whereas star representsthe overexpression of NALCN. The relative expression data were analyzed by the 2^−ΔΔCq^ method. B2M was used as an internal control. (**b**) Correlation analysis of NALCN mRNA expression and NALCN promoter methylation was performed in the same pairs of tissuesamples. mRNA expression of NALCN and hypermethylation of NALCN were evaluated using fold change and threshold was defined as a fold-change > 2. (**c**) Methylation and mRNA expression status of NALCN in NSCLC pair tissues. The red color represents the hypermethylation or overexpression of NALCN, while the green color represents hypomethylation or underexpression.

We quantified the mRNA expression of *NALCN* in a training group of 22 pairs from NSCLC tissues and the adjacent non-cancerous tissues by real-time RT-PCR. *NALCN* was expressed in all tissues, and its expression was normalized with respect to B2M gene expression using the relative quantification approach described by Livak and Schmittgen [25]. We found that the mRNA level of *NALCN* was underexpressed in 12 of 22 (54.5%), NSCLC tissues as the expression of *NALCN* was reduced in tumor samples comparedto the corresponding adjacent tissue. More specifically, the relative expression of *NALCN* (expressed as 2^−ΔΔCt^) was significantly higher (*p* = 0.009) in the tissues of 10 NSCLC patients who were relapse-free (mean = 40.7 ± 23.2) than in the 12 patients who had relapsed during the follow-up period (mean = 6.8 ± 1.8) (Figure 3a). In addition, NALCN was underexpressed in the vast majority of patients who had relapsed (9/12 (75%)), while only 3 out of 10 patients had not relapsed underexpressed *NALCN*.

The methylation status of the *NALCN* gene promoter was further investigated in 11 of the 22 tissue samples for which DNA was available. DNA methylation of the promoter of NALCN was detected in all tissues tested, whether cancerous or not. Hypermethylation of the promoter of the *NALCN* gene was detected in 6 of 9 (66.7%) patients with recurrence and in none of the recurrence-free NSCLC patients. Next, we analyzed the correlation between DNA methylation and mRNA expression using the Spearman correlation test and found that there was no statisticallysignificant correlation betweenthe methylation level of NALCN andthe corresponding mRNA expression (*p* = 0.364, r = −0.304, Figure 3b). Finally, there was a correlation between over- or underexpression of mRNA levels and under- or hypermethylation in 9 out of 11 paired tissue samples tested (Figure 3c).

**Figure 4:**
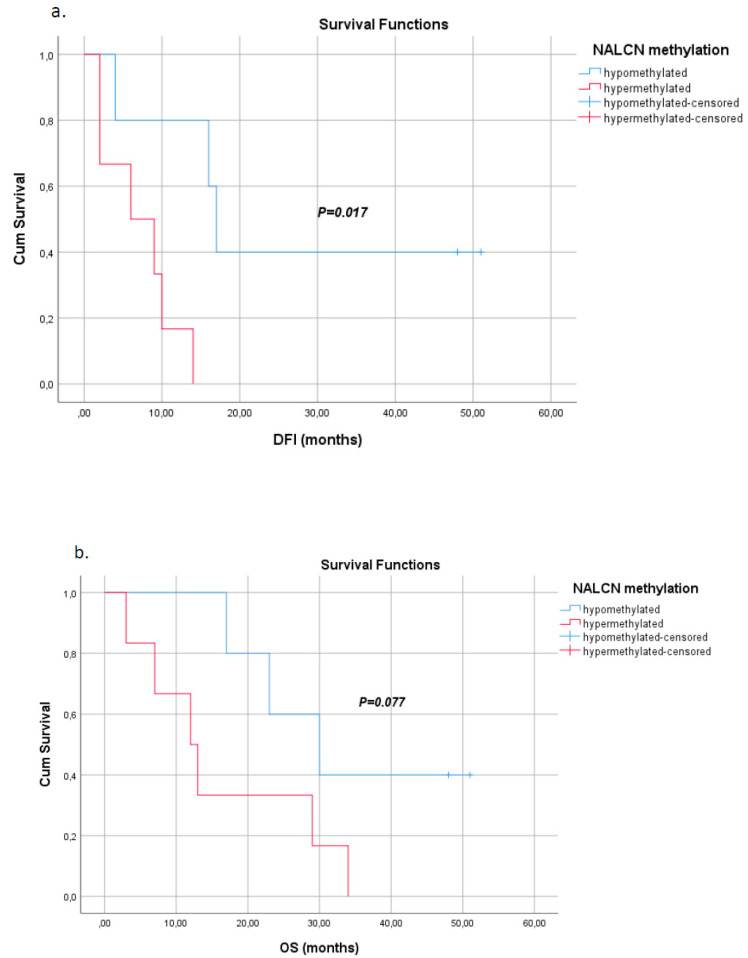
Kaplan–Meier estimates of (**a**) disease-free interval (DFI) in months for NSCLC patients withrespect to NALCN promoter methylation status in tumor tissues (*p* = 0.017), (**b**) overall survival (OS) in months for early NSCLC patients withrespect to NALCN promoter methylation status in tumor tissues (*p* = 0.077).

Kaplan–Meier analysis indicated that NALCN promoter hypermethylation was significantly correlated with worse disease-free intervals (DFIs) (*p* = 0.017, Figure 4a), while itwas not significantly associated with overall survival (OS) (*p* = 0.077, Figure 4b), although it seems to be a trend.

### 3.3. NALCN Methylation in Plasma ctDNA of NSCLC Patients

**Figure 5:**
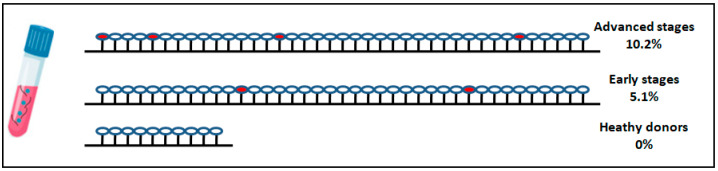
DNA methylation of NALCN gene promoter in plasma cfDNA of patients diagnosed with advanced NSCLC, early-stage NSCLC, and healthy donors. Each cycle represents a patient sample while red cycle represents the hypermethylation of NALCN.

The methylation status of the NALCN promoter was evaluated in ctDNA isolated from plasma of early and advanced NSCLC (validation group) and 10 healthy individuals. Promoter methylation of NALCN was observed in 2 (5.1%) of 39 patients with early NSCLC, 4 (10.2%) of 39 patients with advanced NSCLC, and none (0%) of 10 healthy individuals (Figure 5). Both early-stage patients relapsed and died during the follow-up period of 40 months and the histological type of tissues were adenocarcinoma whereas patients with advanced disease were adenocarcinomas and at the time of diagnosis suffered from nearby and distant lymph node and liver metastasis. All patients died from disease progression and presented a progression-free survival time of 3, 4, 6, and 4 months, respectively.

## 4. Discussion

The current study describes for the first time the existence and prognostic significance of NALCN promoter methylation in NSCLC. Our analysis was performed in cell lines treated with azacitidine and in clinical samples consisting of NSCLC tissue pairs and ctDNA from the plasma of patients with early and advanced NSCLC.

Gene expression of NALCN has been detected in various types of human cancers [24,26]. NALCN was underexpressed in most cancers [24] and was enriched with non-synonymous mutations in gastric, colorectal, lung, prostate, head, and neck cancers [27,28]. It has been suggested that the SNP rs9557635 in the genomic regions of the NALCN gene is associated with advanced NSCLC [29], while Rahrmann et al. have shown that in gastric cancer, deletion of NALCN in mice increases the number of circulating tumor cells and distant metastasis in mice [22]. Mutations of NALCN occur with a similar frequency to TP53 in human cancers, suggesting that NALCN may act as a tumor suppressor [30]. However, there are few studies that have investigated the regulation of NALCN expression by methylation.

He et al. have shown by extensive application of the UALCAN portal that the methylation level of the promoter of NALCN is significantly higher in the vast majority of cancers, including lung cancer, while it is lower in pheochromocytomas and paragangliomas compared to normal tissues [24]. These results support our findings, as according to our results, *NALCN* mRNA levels were significantly higher in the A549 cell line treated with AZA, while there was no effect in the other cell lines tested. The above results suggest that methylation of the promoter of NALCN is negatively related to its mRNA expression and the downregulation of *NALCN* in lung cancer cells is due to methylation of the promoter.

Our results clearly indicate that NALCN is highly methylated in NSCLC tumor tissues, and there was a strong correlation between overexpression or underexpression of its mRNA levels and hypo- or hypermethylation of *NALCN* in 22 of the tissues examined. Moreover, NALCN was methylated in both cancer tissues and adjacent tissues, suggesting that loss of NALCN expression through methylation mechanisms is an early event in NSCLC tumorigenesis [31]. Interestingly, in alimited number of the 11 pairsof tissues, hypermethylation of the NALCN gene promoter was detected in 6 of 9 (66.7%) patients who relapsed, and in none of the two relapse-free NSCLC patients. This finding should be further tested in a large and well-defined patient cohort for the better evaluation of NALCN as a prognostic biomarker.

Numerous other methylated gene promoters detected in ctDNA have been proposed, either individually or in a panel [9], for the detection of lung cancer [16,32]. We report here for the first time the detection of aberrant methylation of the NALCN gene promoter in plasma ctDNA of advanced or early-stage NSCLC patients. According to our results, NALCN was methylated at a lower percentage in the plasma ctDNA of early-stage NSCLC patients (5.1%) than in the plasma ctDNA of advanced-stage NSCLC patients (10.2%). This is consistent with other studies showing that differences in DNA methylation patterns reflect the stage of tumor development [33]. However, due to the limited number of samples, the accuracy of NALCN promoter methylation as a prognostic or diagnostic biomarker in the plasma of lung cancer patients is expected to improve with the use of a sufficient number of samples.

Finally, Kaplan–Meier analysis revealed that the incidence of relapse was higher when NALCN was hypermethylated in primary tissue than in patients in whom the gene promoters were hypomethylated. However, the prognostic significance of NALCN promoter hypermethylation in ctDNA of operable NSCLC or metastatic patients was not evident in our patient cohort.

To our knowledge, fewstudies have focused on the methylation status of NALCN in NSCLC. Based on the above, the detection of NALCN promoter methylation in NSCLC tumor tissue provides important prognostic information for NSCLC patients, while we strongly believe that NALCN promoter methylation in plasma ctDNA should be further evaluated and validated as a non-invasive circulating tumor biomarker in a large and well-defined patient cohort.

## 5. Conclusions

The present study is the first to investigate the prognostic significance of NALCN promoter methylation in NSCLC. Azacitidine-treated cell lines and clinical samples were analyzed, including paired NSCLC tissue and ctDNA from the plasma of patients with early and advanced stages of the disease. NALCN was found to be hypermethylated in NSCLC tissues. Methylation of the NALCN promoter was more frequent in the plasma CTDNA of advanced-stage NSCLC patients than in early-stage patients, but this needs to be further investigated and validated. The study shows that while NALCN promoter methylation in NSCLC tissues provides valuable prognostic information, further research is needed to validate its potential as a non-invasive biomarker in plasma CTDNA in a larger cohort of patients.

## Figures and Tables

**Table 1:** Clinicopathological Characteristics of NSCLC Patients.

		Pair Tissues from Early Stage NSCLC Patients	cfDNA from Early Stage NSCLC Patients	cfDNA from Advantaged Stage NSCLC Patients
**Gender**	*Male*	63.6%	56.4%	85.7%
	*Female*	36.4%	43.6%	14.2%
**Type**	*Adenocarcinoma*	27.3%	46.2%	67.8%
	*Squamous cell carcinoma*	18.2%	53.8%	32.1%
	*N/A*	54.5%	-	-
**Stage**	*IA*	0.4%	25.6%	-
	*IIA*	35.8%	5.1%	-
	*IIIA*	31.7%	15.4%	-
	*IB*	18.1%	20.5%	-
	*IIB*	13.6%	23.1%	-
	*IIIB*	0.4%	7.7%	-
	*IV*	-	-	100.0%
	*N/A*	-	2.9%	-
**Smoking status**	*Yes*	13.6%	71.8%	60.7%
	*No*	36.4%	28.2%	39.2%
	*N/A*	50.0%	-	-

## Data Availability

The data presented in this study are available on request from the corresponding author.

## Supplementary Materials

The following supporting information can be downloaded at: https://www.mdpi.com/article/10.3390/biom14121514/s1, Figure S1: Analytical specificity of the *NALCN* real-time MSP; Figure S2: Analytical sensitivity of the *NALCN* real-time MSP; Table S1: Amplification reaction mixture and run profile for NALCN expression; Table S2: Amplification reaction mixture and run profile for NALCN; Table S3: Amplification reaction mixture and run profile for NALCN promoter methylation.
