# Supplementary material for: NALCN Promoter Methylation as a Biomarker for Metastatic Risk in a Cohort of Non-Small Cell Lung Cancer Patients
Source: Biomolecules. 2024 Nov 27;14(12):1514. doi: 10.3390/biom14121514 (PMC11673096; doi:10.3390/biom14121514)
Supplement: Supplementary file 1 [file biomolecules-14-01514-s001.zip › biomolecules-3240398-Tables.pdf]

Supplementary Table S1: Primers & probes for *NALCN* expression and promoter methylation

NALCN EXPRESSION	
Forward primer	5'-TGAAATATGGGGAGAATATTAACAGG-3'
Reverse primer	5'-TACAAAACGGAGGCTGAACCA-3'
**NALCN PROMOTER METHYLATION**	
Forward primer	5'-TTTTTTTAGTTTGTTCGGTGGCG-3'
Reverse primer	5'-CGCCCAAAACGAACGAAA-3'
Probe	5'-HEX-AGTGCGTTGTTTGAGTTGAGTCGT-BHQ-3'

Supplementary Table S2: Amplification reaction mixture and run profile for *NALCN*

Reagents	Volume per reaction (µL)
H_2O	3.8
PCR Synthesis buffer (5x)	2
$MgCl_2$ (25mM)	1.2
dNTPs (10mM)	0.2
BSA (0.1 µg/µL)	0.5
Forward primer (10mM)	0.2
Reverse primer (10mM)	0.2
LC Green Plus Dye	1
Hot-Start DNA Polymerase	0.1

Hold (1 cycle)at 95°C for 5 min, **Cycling (45 cycles)**1) 95°C for 10s 2) 62°C for 15s 3) 72°C for 15s, **Melt** from 72°C to 95°C at 0.3°C/s

Supplementary Table S3: Amplification reaction mixture and run profile for *NALCN* promoter methylation

Reagents	Volume per reaction (µL)
H_2O	2.94
PCR Synthesis buffer (5x)	3
$MgCl_2$ (25mM)	1.2
dNTPs (10mM)	0.2
BSA (0.1 µg/µL)	0.3
Forward primer (10mM)	0.3
Reverse primer (10mM)	0.3
LC Green Plus Dye	1
Hot-Start DNA Polymerase	0.1

**Hold (1 cycle)**at 95°C for 2 min, **Cycling (45 cycles)** 1) 95°C for 10s 2) 63°C for 20s 3) 72°C for 20s,**Melt** from 55°C to 95°C at 0.1°C/s
