# Supplementary figures and images for: NALCN Promoter Methylation as a Biomarker for Metastatic Risk in a Cohort of Non-Small Cell Lung Cancer Patients
Source: Biomolecules. 2024 Nov 27;14(12):1514. doi: 10.3390/biom14121514 (PMC11673096; doi:10.3390/biom14121514)

Supp.  
Figure S1

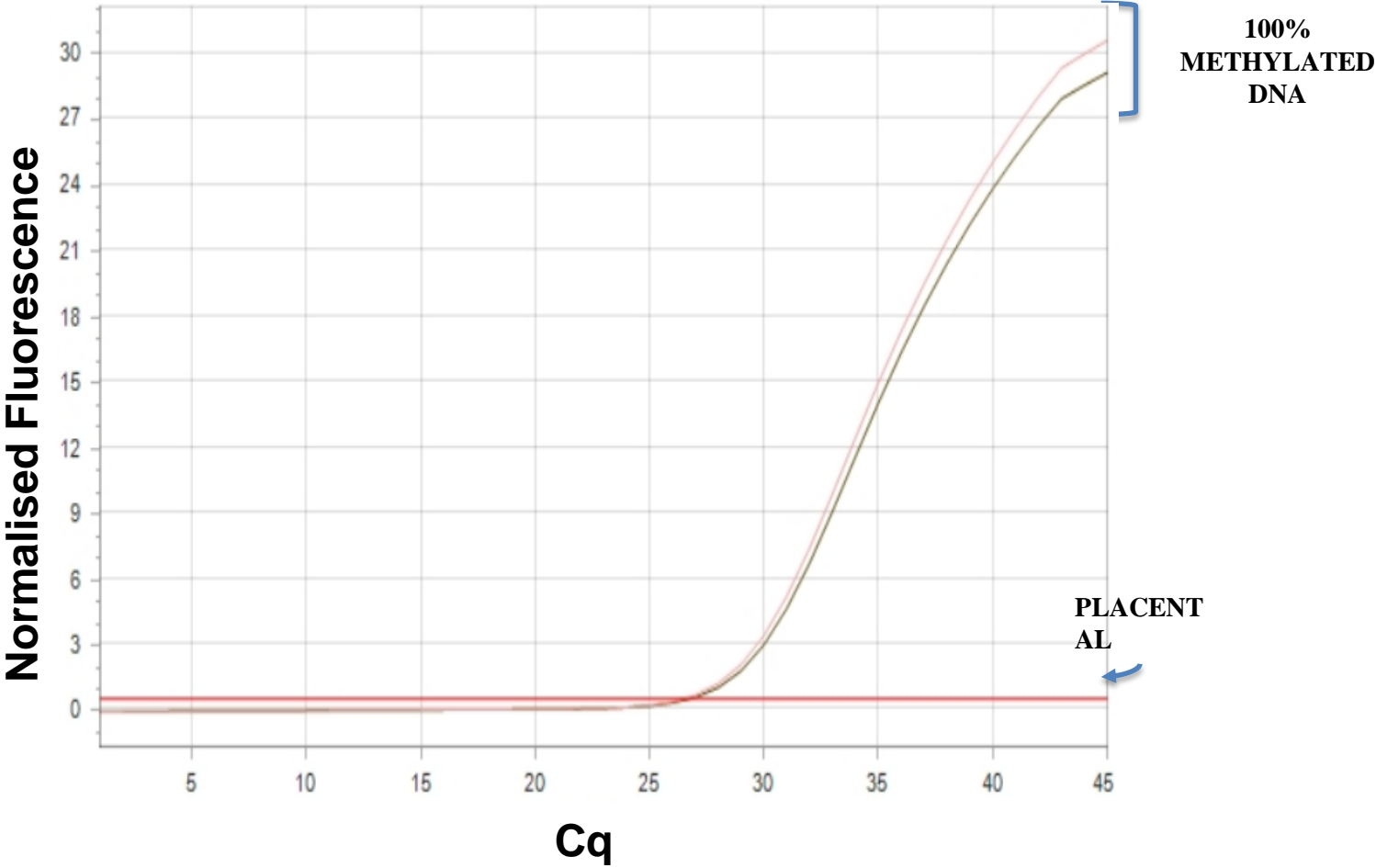

\_\_\_\_\_

Supp. Figure S2

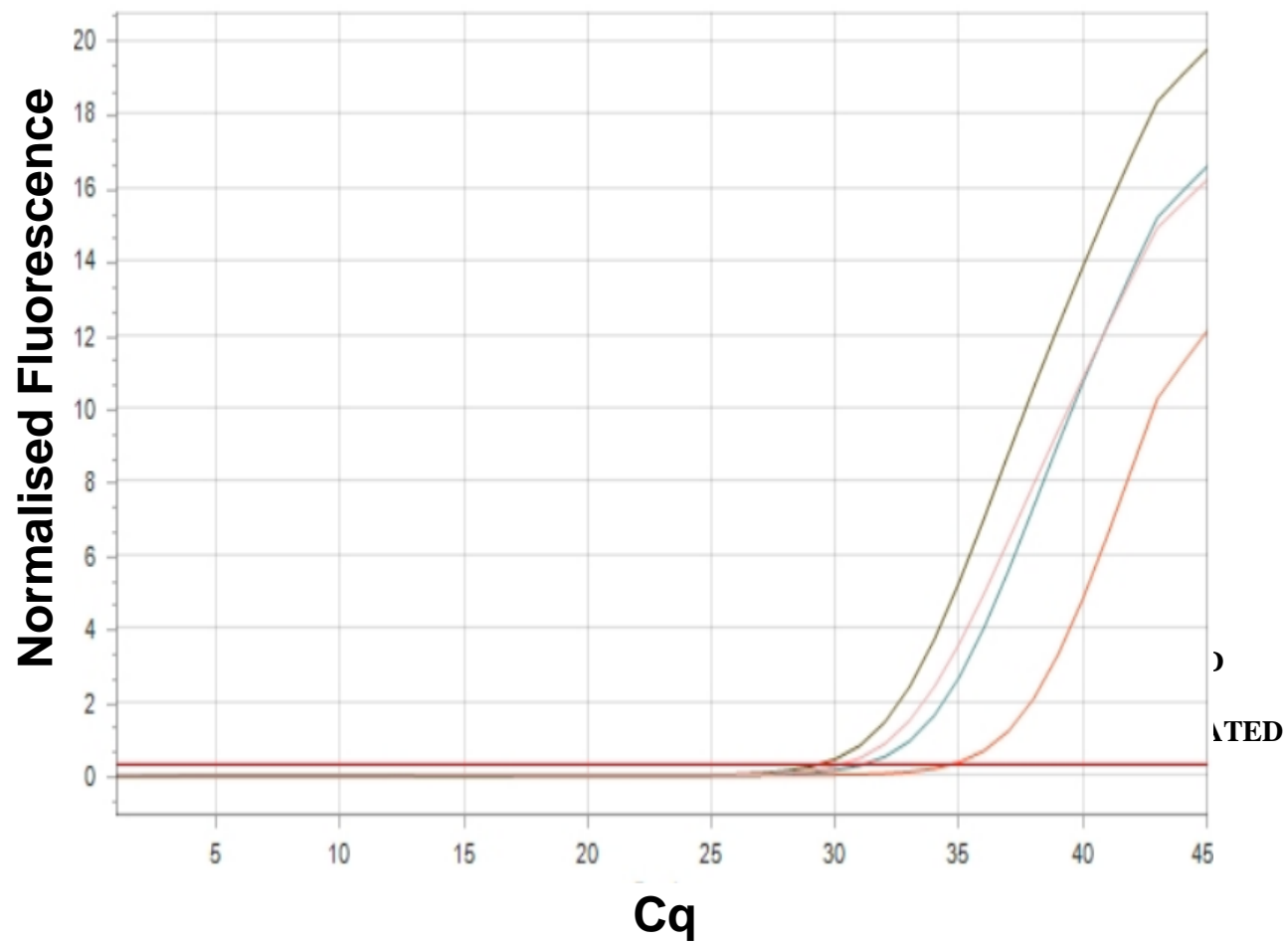

Supplement: Supplementary file 1 [file biomolecules-14-01514-s001.zip › biomolecules-3240398-figures.pdf]
